# A study to examine the frequency of admissions to ICU in relation to the hour of admission

**DOI:** 10.1186/2197-425X-3-S1-A139

**Published:** 2015-10-01

**Authors:** A Gupta, P Morgan

**Affiliations:** Surrey and Sussex Hospitals Trust, Intensive Care, Redhill, United Kingdom

## Introduction

In order to determine staffing level allocations following the expansion of our intensive care unit we conducted a preliminary retrospective study that looks at the number of admissions at each hour of the day. We also correlated this to patient outcome as we felt this may also influence the chosen staffing levels throughout the day.

## Objectives

To evaluate the frequency of acute medical admissions to SASH ICU at each hour of the day.

## Methods

All patients included in our sample population were categorised on the hour of admission to SASH ICU. The number of admissions at each hour were calculated and correlated with accompanying mortality data.

Retrospective study.

Search criteria were inputted into the ICU ward watcher database. We excluded elective ICU/HDU surgical admissions from the patient population and included patients between the ages of 17 and 98 years of age.

Sample population size: 9,092 patients admitted to SASH ITU. Our data looks at total number of patients meeting the aforementioned criteria between 29/12/1992 to 25/12/2014.

## Results

**Figure 1:**
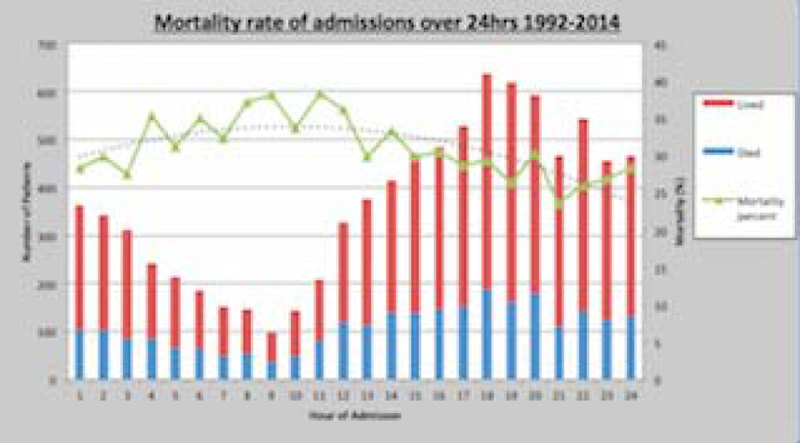
**Mortality rate of admissions over 24hrs 1992-2014.**

The data (figure 1)shows that most admissions occur in the late afternoon and throughout the evening. These numbers then tail off in the early hours of the morning.

Interestingly, it appears that frequency of ICU admissions at these times relates paradoxically to outcome. When compared to admissions in the early morning, there is a worse outcome a lower number of admissions.

## Conclusions

There is significant variation that patients are admitted to ICU over a 24-hour period. As the day progresses into the afternoon and evening, emergency medical admissions to ICU also increase (peaking between 17:00 and 20:00). This maybe due to increased bed movements at this time related to medical discharges elsewhere within the hospital[1]. Nonetheless, further work must be carried out to examine the underlying causes for this trend.

Interestingly, this preliminary study also indicates that the number of admissions can have a paradoxical inverse relationship with outcomes. The underlying cause for both these results must be investigated further.

Future work will look at the extent to which out-of-hours healthcare professional staffing levels relates to patient outcome and delineate specific factors that may have led to delayed admissions to the intensive care from the time of referral.
